# Discriminating between Interstitial and Circulating Leukocytes in Tissues of the Murine Oral Mucosa Avoiding Nasal-Associated Lymphoid Tissue Contamination

**DOI:** 10.3389/fimmu.2017.01398

**Published:** 2017-10-30

**Authors:** Peter D. Bittner-Eddy, Lori A. Fischer, Andy A. Tu, Daniel A. Allman, Massimo Costalonga

**Affiliations:** ^1^Division of Periodontology, Department of Developmental and Surgical Sciences, School of Dentistry, University of Minnesota, Minneapolis, MN, United States

**Keywords:** interstitial leukocytes, circulating leukocytes, *Porphyromonas gingivalis*, oral mucosa, nasal-associated lymphoid tissue, memory T cells, periodontitis

## Abstract

Periodontitis is a chronic inflammatory response to a microbial biofilm that destroys bone and soft tissues supporting the teeth. Murine models of periodontitis based on *Porphyromonas gingivalis* (*Pg*) colonization have shown that extravasation of leukocytes into oral tissue is critical to driving alveolar bone destruction. Identifying interstitial leukocytes is key to understanding the immunopathogenesis of periodontitis. Here, we describe a robust flow cytometry assay based on intravenous FITC-conjugated anti-mouse CD45 mAb that distinguishes interstitial leukocytes in the oral mucosa of mice from those circulating within the vasculature or in post-dissection contaminating blood. Unaccounted circulating leukocytes skewed the relative frequency of B cells and granulocytes and inflated the numbers of all leukocyte cell types. We also describe a dissection technique that avoids contamination of oral mucosal tissues with nasal-associated lymphoid tissues (NALT), a B cell rich organ that can inflate leukocyte numbers at least 10-fold and skew the assessment of interstitial CD4 T cell phenotypes. Unlike circulating CD4 T cells, interstitial CD4 T cells were almost exclusively antigen-experienced cells (CD44^hi^). We report for the first time the presence of antigen-experienced *Pg*-specific CD4 T cells in NALT following oral feeding of mice with *Pg*. This new combined flow cytometry and dissection approach allows identification of leukocytes infiltrating the connective tissues of the murine oral mucosa and avoids confounding analyses of leukocytes not recruited to inflamed oral mucosal tissues in disease conditions like periodontitis, candidiasis, or sialadenitis.

## Introduction

The mouth harbors a species-rich microbiome that includes microorganisms with pathogenic and invasive potentials and the ability to form biofilms on hard surfaces like teeth (1, 2). Inflammatory responses to these microorganisms are surprisingly rare due in part to epithelial shedding and the generally tolerogenic nature of the oral mucosa (3). In mice, similar to man, the oral mucosa consists of discrete buccal, sublingual, sulcular, and gingival surfaces that comprise a stratified squamous epithelium of varying thickness overlaying a lamina propria (4). Destructive inflammatory diseases can occur when microorganisms breach the sulcular epithelium around teeth. As one such disease, periodontitis is triggered by a community of symbiont microorganisms whose relative proportions are altered by a handful of microorganisms, so called “keystone pathogens,” which are capable of altering the nutrient foundation of the community itself (5–7). This changed nutritional environment fosters the growth of pathobionts that ultimately stimulate the extravasation and infiltration of the leukocytes into the interstitium of the gingival tissues. This cellular infiltrate consists of both innate and adaptive immune cells including neutrophils, monocytes/macrophages, conventional α/β and γ/δ T cells, B1 and B2 cells, NK T cells, and dendritic cells (8–11). Though recruited to fight the invading pathobionts, the cellular infiltrate also triggers osteoclastogenesis leading to alveolar bone destruction as well as irreversible loss of the connective tissue attachment that holds teeth into their socket (12, 13).

Understanding the immunopathogenesis of periodontitis is critical to strategies that seek to prevent, treat, or predict the occurrence and progression of this disease. Mouse periodontitis models based on the keystone pathogen *Porphyromonas gingivalis* (*Pg*) have been instrumental in expanding our knowledge of this disease (14–19). However, a number of these studies have been limited by an inability to unambiguously identify, quantify, and analyze leukocytes that have extravasated into gingival tissues in response to pathogen challenge (10, 20–23). In mice the assessment of interstitial immune cells in oral mucosa can be confounded by cells coming from three sources: (i) contamination from nasal-associated lymphoid tissue (NALT), which lay directly beneath the anterior hard palate near the marginal gingiva of first molars, (ii) contamination from blood released during dissection, or (iii) from blood trapped within the tissue vasculature. Interstitial leukocytes have very different phenotypes and functions compared to their counterparts in systemic circulation and in lymphoid tissues like NALT (24–27). Therefore, it is critical to distinguish these different leukocyte populations. Perfusion of animals postmortem with saline has been used to remove leukocytes from tissue vasculature with some success. However, perfusion is time consuming and is not effective in removing all cells residing in capillaries leading to the potential to misinterpret data (28). Other techniques to distinguish interstitial from circulating leukocytes have used the intravenous delivery of fluorochrome-conjugated anti-mouse mAbs into mice. This technique has been successfully applied to identify and analyze interstitial, marginated, or alveolar leukocytes in the lung of mice under homeostatic or pathogen-induced injury and inflammation (28–31). Monoclonal antibodies that specifically target CD8 T cells (28, 30, 32) or neutrophils (29) have been used in addition to mAbs that target all leukocytes (31). This technique, however, has not been used in mice for the specific purpose of identifying leukocytes that reside solely within the interstitium of the oral mucosa.

Here, we describe a simple and robust flow cytometry-centered assay that distinguishes interstitial leukocytes in the oral mucosa from those found within the tissue vasculature and in contaminating extravascular blood. This method is based on the intravenous delivery of an anti-mouse mAb specific for CD45, a pan-leukocyte marker. We also describe a dissection technique that avoids contamination of oral mucosal tissues with NALT cells. Using this combined approach allows the unambiguous identification of leukocytes residing in the interstitium of the oral mucosa at homeostasis or following gingival inflammation induced by pathogens like *Pg*.

## Materials and Methods

### Mice

All animal experiments were reviewed and approved by the Institutional Animal Care and Use Committee of the University of Minnesota (Protocol ID # 1511-33178A) and performed on 6- to 8-week-old male and female littermates. C57BL/6J and BALB/cJ mice were bred in house from stocks originally purchased from The Jackson Laboratory (Bar Harbor, ME, USA). Mice were housed in microisolator cages with food and water *ad libitum* in a specific pathogen-free animal facility in compliance with the Association for Assessment and Accreditation of Laboratory Animal Care at the University of Minnesota.

### Infection of Mice with *Pg*

*Porphyromonas gingivalis* strain ATCC 53977 (A7A1-28) was originally obtained as a gift from Dr. P. Baker (Bates College, Lewiston, ME, USA) (16). *Pg* was grown anaerobically at 37°C for 7–14 days in 5% CO_2_/10% H_2_/85% N_2_ on Todd–Hewitt broth–blood agar plates, supplemented with 5.0 mg/ml hemin, 0.5 mg/ml menadione, and 25 µg/ml gentamycin. For analysis of the NALT, mice were inoculated with *Pg* or vehicle alone by oral gavage every 3/4 days and sacrificed on day 28 as previously described (17, 33).

### Intravenous Delivery of Pan-Leukocyte Monoclonal Antibody

FITC-conjugated rat anti-mouse CD45 monoclonal antibody (eBioscience San Diego, CA, USA; clone 30-F11) was prepared for intravenous injection by diluting 1.25 µg of the antibody in 200 µl of sterile PBS per mouse. Mice were anesthetized through controlled inhalation of isoflurane, laid on their sides and the PBS/antibody solution injected into the retro-orbital cavernous sinus using a disposable 1-ml tuberculin syringe. Animals were returned to their cage, monitored for full recovery and then sacrificed through CO_2_ inhalation at 0.5, 3, 30, and 60 min and the carcasses immediately placed on ice to limit diffusion of the mAb. When required, blood (100–150 µl) was collected by facial vein puncture immediately postmortem. Typically mice were injected in pairs with no more than three pairs in a given experiment to minimize the potential variability related to passage of time between anti-CD45 mAb injection and tissue harvest. Each pair of mice were processed within 1 h. Small numbers of mice is important because even though the mAb is no longer circulated after euthanasia, it has the potential to bind to leukocytes postmortem.

### Separate Dissection of Oral Mucosa and NALT

Mouse fur is wetted with PBS and heads severed from the carcass on a wet towel. The sagittal separation of the two hemimandible is performed with a single antero-posterior cut using a small scissor (G. Tierman & Co., Hauppauge, NY—Cat #105-422). Invariably, the tongue moves to one side during this cut. The tongue is pealed caudally and completely excised with two cuts by leaning the scissors toward the lingual side of the mandible between the tongue and the medial side of each hemimandible. The tongue is removed. With two large T pins the base of the skull is penetrated caudocranially and secured on a polystyrol foam dissecting board with maxillary (MX) teeth and palate facing upward under a dissecting microscope at 20× magnification.

**Figure 1:**
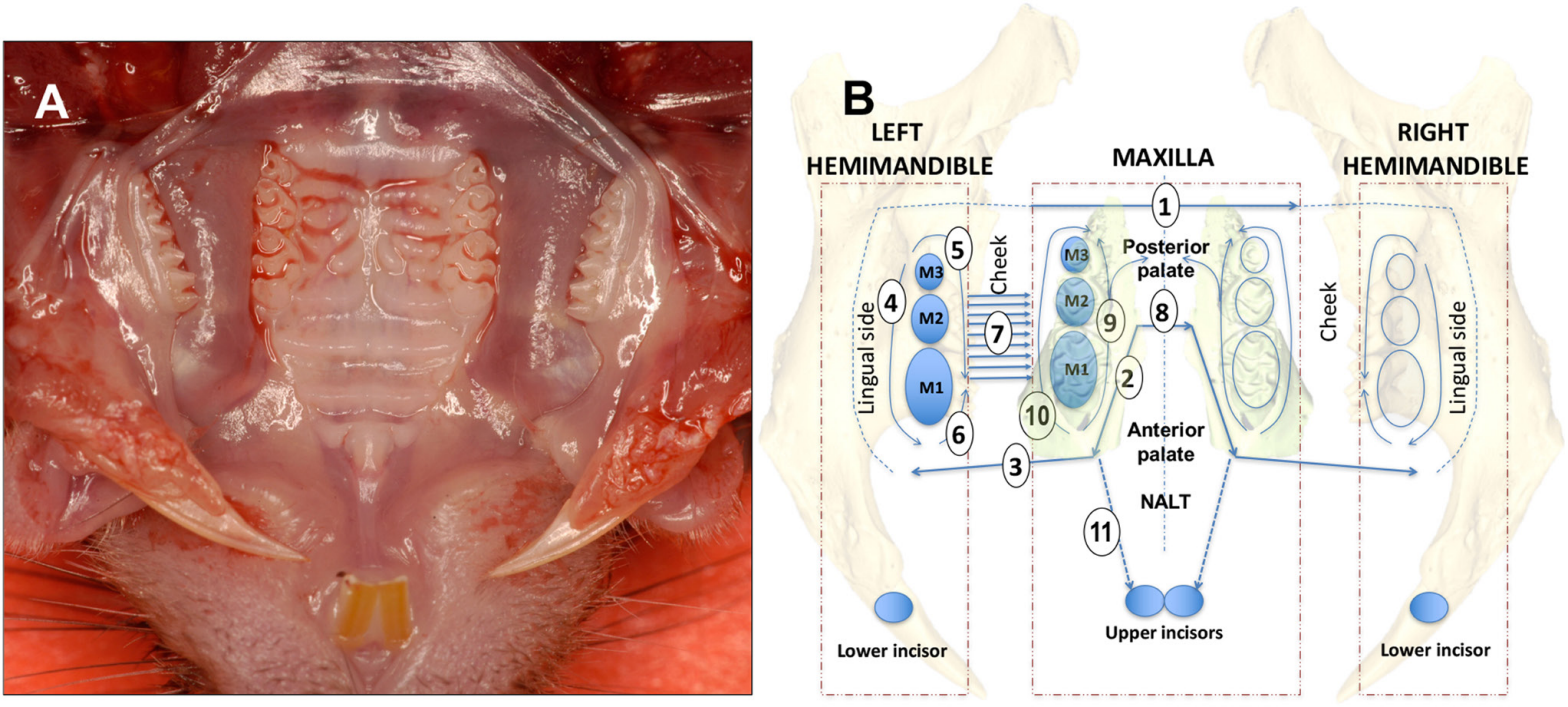
Dissection scheme of oral mucosa and nasal-associated lymphoid tissue (NALT). **(A)** Occlusal view of the mouth of a C57BL/6J mouse showing upper molars and hard palate prior to removal of the oral mucosa. **(B)** For a right-handed operator with the incisor teeth facing the operator the dissection steps are numbered sequentially beginning with the first of four cuts made using the side blade of an 18-G needle [1, 2, 3, and 8 (straight arrow lines)]. Full thickness flap reflection steps are 4, 5, 6, 7, 9, and 10 [curving arrow lines]. The left side is dissected first followed by the right side with complete excision of the oral mucosa. The NALT is dissected last with the incision of step 11 and reflection of the anterior palate bound to the floor of the nose where the NALT sits. See Section “Materials and Methods” text for a more detailed description. Mouse teeth are depicted as blue ovals and upper and lower molars are numbered M1, M2, and M3.

**Figure 2:**
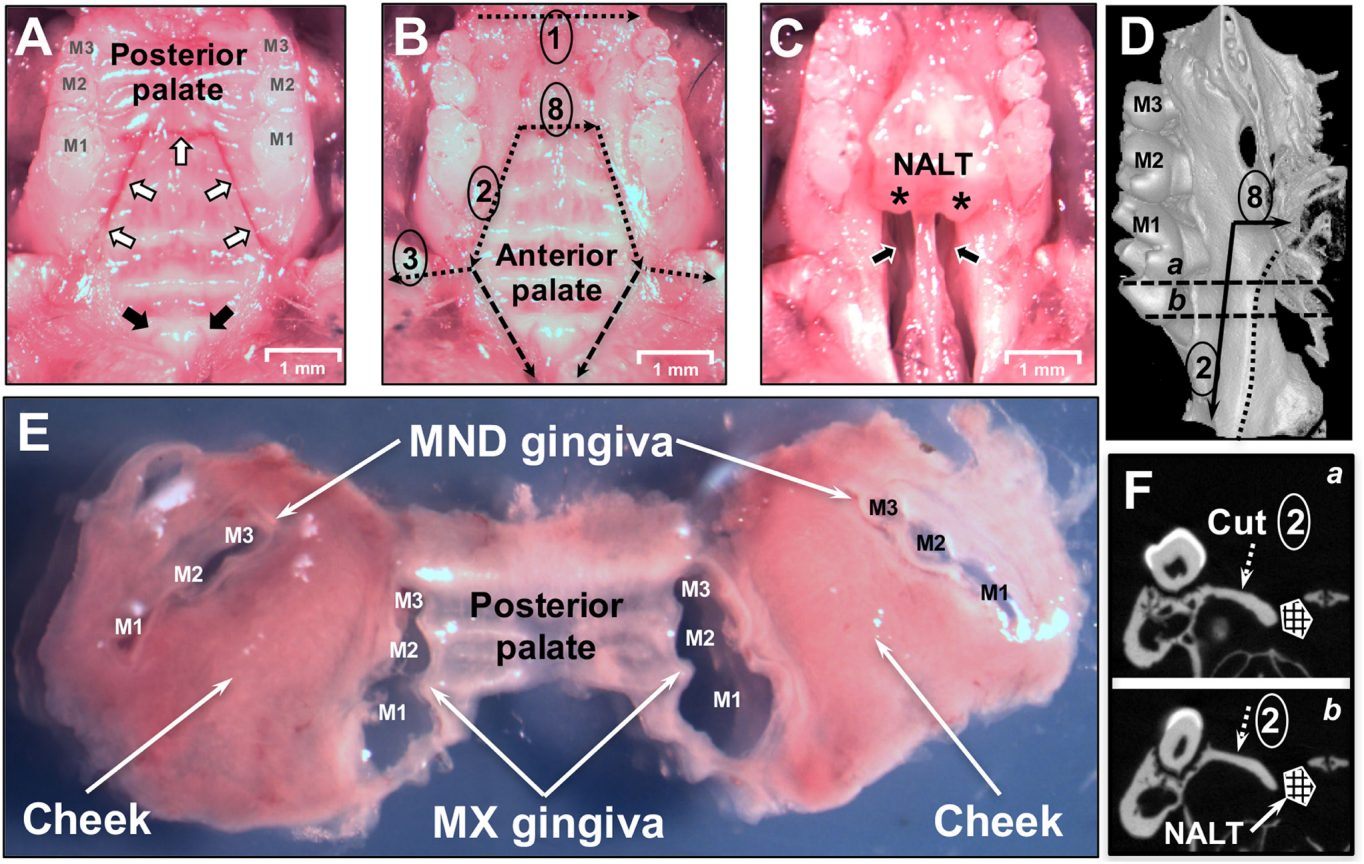
Dissection of oral mucosa. **(A)** Occlusal view of the maxilla of a C57BL/6J mouse showing the three upper molars (M1, M2, M3) and hard palate prior to removal of the oral mucosa. White arrows indicate incision lines made in the hard palate down to the maxillary (MX) bone. Black arrows indicate the two entrances of the patent nasopalatine canal located just posterior to the two incisors. **(B)** Dissection cuts 1, 2, 3, and 8 are indicated by dotted black lines. Marginal gingiva and part of the posterior hard palate have been removed. The anterior hard palate is left *in situ* and is visible. Segmented black lines are the dissection cuts to harvest the anterior hard palate that includes the nasal-associated lymphoid tissue (NALT). **(C)** Anterior hard palate remnant peeled back to reveal the underlying nasal passages (black arrows) and intact left and right NALT which are marked by asterisks (*). **(D)** Micro Computed Tomography (MicroCT) 3D reconstruction showing the medial side of the left hemimaxilla with the three molars. Black arrows show the incision lines #2 and #8. Dotted line shows the boundary of the NALT area. Segmented line “a” and “b” show the cutting planes displayed in panel [**(F)** a,b]. **(E)** Dissected oral mucosa including posterior hard palate, mandibular (MND) and MX marginal gingiva, and bilateral mucosa of the cheeks. **(F)** MicroCT section on a frontal plane crossing the first molar in the two parallel planes (a,b) shown in panel **(D)**. The segmented white arrow indicates the MX alveolar bony shelf where cut #2 lands. NALT location is indicated by the hashed box.

The hemimandibles are rotated outward as to open a book and the initial cut penetrates with scissors the medial surface of the mandibular (MND) ramus cutting the digastric, medial pterygoid and temporal muscles and freeing the condile from the MND/glenoid fossa (Figure 1A). The palate is now well visible for the dissection of the palatal gingival, while avoiding the NALT. From this point forward, all incisions are performed with the side of the cutting edge of an 18-G needle. The first cut [Figure 1B—step (1) of a diagram that shows this and all subsequent cuts, including flap reflection steps] separated the mucosa of the soft palate from the posterior hard palate cuting across the midline. The next two cuts are critical and separate the NALT from the gingiva of the first molar [Figure 1B (2) and Figures 2B,D,F, a,b]. The blade orientation generates an internal bevel incision (34, 35) from mesial MX second molar (M2) along the palatal side of the MX first molar (M1) to end mesio-buccally of MX first molar beyond the mucogingival junction into the buccal fold on left and right sides [Figure 1B (2) and Figure 2B]. Critically, this incision lands on MX alveolar bony shelf laying between the roots of the first molar tooth and the NALT housing (Figures 2D,F). The NALT lays just cranially to the surface of the mid and anterior hard palate (Figure 2C) (36). These two incisions are extended laterally into the inner surface of the cheek to reach anterior to the mesial surface of the first MND molar along a ligament that connects the maxilla to the mandible [Figure 1B (3)]. Next, the remaining lingual mucosa of the floor of the mouth is separated from the mylohyoid muscle, reflected coronally to dissect the marginal gingiva along the lingual surface of the hemimandible [Figure 1B (4)]. Starting at the distal surface of the third MND molar (M3), the MND buccal gingiva is reflected up to the mid-buccal of the first molar [Figure 1B (5)] to meet the buccal flap reflection that was simultaneously initiated at the mesial surface of the first molar [Figure 1B (6)]. Now that the gingiva around the mandibibular teeth is completely reflected, we proceed with the blunt dissection of the mucosa of the cheek to reach the attached gingiva of the buccal surface of the maxilla [Figure 1B (7)]. The two palatal cuts are now connected across the midline at the level of the mesial surface of the second molar [Figure 1B (8), Figure 2B (8), and Figure 2D (8)]. The mesio-palatal gingiva of the MX first molar is then lifted with the tip of the 18-G needle and the palatal gingiva is reflected up to the mesial surface of the third molar [Figure 1B (9)]. The tip of the 18-G needle is now bent by pressing the needle on glass at a 45° angle. The bent tip allows the reflection of the buccal gingiva from mesio-buccal first MX molar to the distal of the third MX molar [Figure 1B (10)]. This incision and reflection scheme allows harvesting of the entire oral mucosa excluding NALT, tongue, and skin of the perioral area.

Nasal-associated lymphoid tissue is subsequently dissected by making two incisions between the mesial area of the MX first molar and the central incisor [Figure 1B (11)]. The anterior hard palate left attached to the maxilla is then easily peeled-off (Figure 2C) and can be processed for tissue disruption.

### Analysis of Leukocytes in Oral Mucosa, Blood, or Cervical Lymph Nodes (CLNs) by Flow Cytometry

Blood samples were treated with ACK lysing buffer (Lonza, Walkersville, MD, USA) prior to analysis. CLNs were harvested by cutting the skin along the midline and pealing it laterally. On each side of the mandible two small anterior LN were identified and harvested just below the inferior border of each hemimandible. A second larger LN was harvested at the posterior edge of the corner of each hemimandible anterior to the sternocleidomastoid muscle. The six CLN were disrupted with the back of a 3-ml syringe plunger over a 70-µm nylon mesh in a 60-mm diameter Petri dish to produce a single-cell suspension that is subsequently filtered through a 70-µm cell strainer (17, 33). Dissected oral mucosa was minced to approximately 2 mm^2^ using scalpel blades and placed in 2 ml of complete EHAA (Life Technologies, Carlsbad, CA, USA) in a 50-ml disposable conical tube. Tissue was further disrupted by a 60-min incubation at 37°C in the presence of 2 mg/ml collagenase D (Roche Diagnostics, Indianapolis, IN, USA) and 1 mg/ml DNase I (Sigma-Aldrich, St. Louis, MO, USA). EDTA was added for the last 10 min to a final concentration of 5 mM to help disrupt Ca^2+^-mediated cell-to-cell interactions. Tissue was mashed and passed through 70-µm cell strainers to remove clumps and debris. Single-cell suspensions from blood, CLNs, or oral mucosa were washed with PBS before being stained with cell viability dye Zombie NIR (BioLegend, San Diego, CA, USA) for 10 min in the dark at room temperature. Fc receptors were blocked using anti-mouse CD16/CD32 antibody (eBioscience; clone 93) before being stained with anti-mouse CD45 mAb conjugated to PE (eBioscience; clone 30-F11). In some experiments, cells were also stained with fluorochrome-conjugated anti-mouse CD3:BV510 (BioLegend; clone 17A2), CD4:PerCp/Cy5.5 (eBioscience; clone RM4-5), CD8:e450 (eBioscience; clone 53-6.7), CD11b:BV650 (BioLegend; clone M1/70), CD44:APC/Cy7 (BioLegend; clone IM7), B220:e450 (eBioscience; clone RA3-6B2), or GR1:AF647 (BioLegend; clone RB6-8C5) mAbs. Cells were acquired on an LSR II flow cytometer (BD Biosciences, San Jose, CA, USA) and fluorescence emissions analyzed with FlowJo software (Tree Star, Ashland, OR, USA).

### Identification of *Pg*-Specific CD4 T Cells in NALT

Nasal-associated lymphoid tissue from C57BL/6J mice was harvested 28 days after sham (vehicle only) or *Pg* inoculation. NALT were mechanically disrupted and single-cell suspensions from a single mouse were prepared using standard methods (36). Cells were incubated with 5–25 nM PE-conjugated pR/Kgp:I-A^b^ tetramer at room temperature followed by a panel of anti-mouse mAbs to identify antigen-experienced CD44^hi^ CD4 T cells, CD8 T cells, and B cells as previously described (33). 1–2 × 10^5^ cells in the lymphocyte gate were counted and acquired on an LSR II flow cytometer and analyzed with FlowJo software.

### Statistical Analysis

Cell frequency was analyzed and plotted using Prism 6 software (GraphPad Software, San Diego, CA, USA) and expressed as mean ± SEM. Means were compared using two-tailed Student *t*-tests. A *p* value < 0.05 was considered significant.

## Results

### Antigen-Experienced *Pg-*Specific CD4 T Cells Can Be Found in NALT

**Figure 3:**
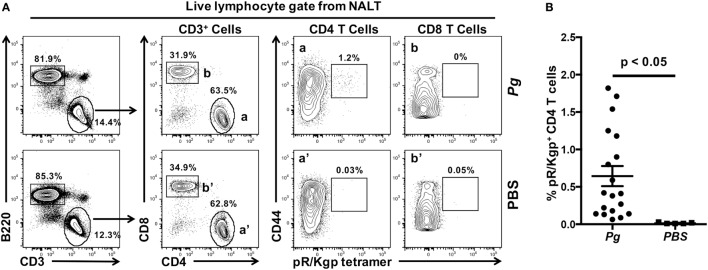
Nasal-associated lymphoid tissue (NALT) harbor *Porphyromonas gingivalis* (*Pg*)*-*specific CD4 T cells. C57BL/6J mice were orally inoculated with *Pg* or vehicle (PBS) every 4 days and on day 28 mice were sacrificed and NALT harvested. Single-cell suspensions were produced and cells were stained with vitality dye Zombie NIR followed by rat anti-mouse CD45, B220, CD3, CD4, CD8, and CD44 mAbs. A pMHCII tetramer reagent (pR/Kgp:I-A^b^ tetramer) was used to identify CD4 T cells that are specific for an epitope found in *Pg*. Cells were counted on an LSRII flow cytometer and analyzed using FlowJo software. **(A)** Representative dot plots of live lymphocytes obtained from the NALT of a PBS or *Pg-*inoculated mouse are shown. Percentage of cells found within each gate is indicated. **(B)** Summary of the % of pR/Kgp-specific antigen-experienced CD4 T cells found in NALTs following PBS or *Pg* inoculation. Data from three experiments is displayed as mean ± SEM. Student’s two-tailed *t*-test was used to compare means.

Nasal-associated lymphoid tissue in mice has been considered to be the equivalent of the adenoids and palatine tonsils in primates (37). The NALT in rodents are strategically located to sample microbes in the air stream coming from the nares (38) and in fluids accessing the nose from the oral cavity through a patent nasopalatine canal (Figure 2A—black arrows) while the rodent is nuzzling, a process of tasting and smelling at the same time while feeding (39–41). Therefore, the nasopalatine canal could easily act as a conduit for oral bacteria to reach the nasal passages (Figure 2C—black arrows). NALT lie directly beneath the hard palate (Figure S1 in Supplementary Material; Figures 2B,C) and act as a major mucosal inductive site that provides both B and T memory cells for local dissemination (36, 42). Using a well-established murine model of periodontitis (15), we have shown previously that oral inoculation of mice with *Pg* resulted in the induction of a robust Th17 response in the CLNs that drain the oral cavity (17). Whether the NALT acts as an inductive site and/or as a source of recirculating memory CD4 T cells specific to *Pg* has not been determined. Our studies show that NALT has the capacity to act as an inductive site for memory CD4 T cells against nasally introduced pathogens (36, 37, 43, 44). To investigate whether oral bacteria prime CD4 T cells in the NALT, mice were orally inoculated with *Pg* for 28 days. Anterior palate/NALT were subsequently removed and single-cell suspensions stained with anti-mouse mAbs and a pMHCII tetramer reagent (pR/Kgp-Tet) to identify antigen-experienced CD4 T cells that are specific to *Pg* epitopes (33). We routinely found NALT to have higher numbers of B cells (B220^+^ CD3^−^) compared to CD4 and CD8 T cells (Figure 3A). As predicted, we discovered significant numbers of antigen-experienced *Pg*-specific CD4 T cells (CD44^hi^ pR/Kgp-Tet^+^) residing in the NALT of mice orally fed with *Pg* (Figure 3B). These cells averaged 0.64% (*n* = 19) of the total CD4 T cell population found in the NALT, and the majority of these cells expressed high levels of CD44 marking them as antigen-experienced T cells.

### Analysis of Leukocytes in the Oral Mucosa Begins with Precise Dissection That Avoids NALT

Our analysis revealed that NALT has the potential to be a significant source of not only contaminating B and T cells, but also of *Pg*-specific CD4 T cells in mice orally inoculated with *Pg*. Due to the close proximity of NALT to the oral mucosa that surrounds the maxilla, isolation of leukocytes that reside in tissue such as the marginal gingiva must start with tissues that are free of cells coming from secondary lymphoid organs such as the NALT. To achieve this objective, we designed a dissection protocol that preserves a strip of hard palate as the physical barrier between NALT and the marginal gingiva (Figures 1 and 3). The critical incision line in the thickness of the hard palate lands on the MX alveolar bony shelf (Figures 2D,F; cut #2). The incision starts from the distal end of the first molars and is extended anteriorly and laterally to reach the gingiva mesial to the first molar (Figure 2A—white arrows). Further incisions allowed the oral mucosa and the posterior hard palate to be lifted away without disturbing tissue that overlays the nasal passages and NALT (Figure 2B). When the anterior hard palate is lifted and peeled back distally, the nasal passages (Figure 2C—black arrows) and the intact NALT organs are revealed (Figure 2C—*). This dissection technique allows the oral mucosa including the cheeks to be harvested as one piece (Figure 2E) without disturbing the underlying NALT that lays on the cranial surface of the anterior hard palate (Figures 1B and 2B,C) (36). After lifting of the oral mucosa as one piece, the NALT was subsequently dissected [Figure 1B (11)], harvested (Figure 2C), and analyzed independently (Figures 3A,B).

**Figure 4:**
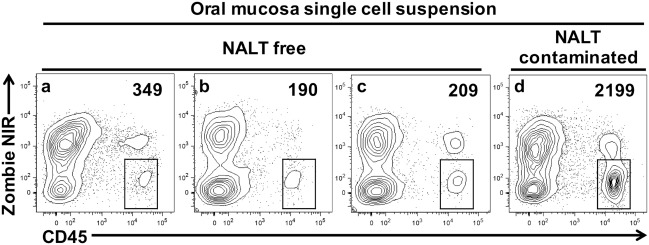
Nasal-associated lymphoid tissue (NALT) contamination results in the over estimation of leukocyte cells within the oral mucosa tissues. Oral mucosa was harvested from the maxilla of C57BL/6J mice, digested with collagenase D, and processed to obtain single-cell suspensions. Cells were stained with vitality dye Zombie NIR followed by rat anti-mouse CD45 mAb conjugated to PE to identify live leukocytes by flow cytometry. Representative dot plots generated by FlowJo analysis are shown. In three of the samples shown **(A–C)**, marginal gingiva was harvested without disruption of the anterior hard palate as outlined in Figures 1 and 2. In a fourth sample **(D)**, incision #2 was medial, landing over the NALT compartment in the anterior hard palate and exposing the oral mucosa to potential NALT contamination. The live leukocyte cell population (Zombie NIR^low^, CD45^+^) is gated and the number of cells found in these gates is given. Comparable results were seen in two other similar experiments.

We next prepared single-cell suspensions from oral mucosa samples and stained them to identify live leukocytes (CD45^+^) by flow cytometry. We compared samples obtained by the dissection method outlined above with samples obtained from mice where the oral mucosa was exposed to NALT contamination through breaching the barrier formed by the hard palate (Figure 4). In the latter mice, we deliberately made incision #2 (Figures 1B and 2B) slightly medial into the hard palate covering the nasal passages. We determined that NALT contamination could be a significant source of contaminating CD45^+^ cells in oral mucosa samples when the dissection did not land on the palatal shelf of the MX alveolar bone (Figure 4d). This faulty dissection of the oral mucosa yielded on average 10 times more CD45^+^ cells (Figure 4 and data not shown) than oral mucosa samples that avoided NALT through the precise dissection procedure outlined in Figures 1 and 2.

### Isolation and Identification of Interstitial Leukocytes in Oral Mucosa

Preparations of leukocytes residing within the tissues of the oral mucosa can be contaminated directly with circulating leukocytes present in the vascular network or indirectly through blood released during dissection. These contaminating sources of leukocytes have the potential to confound the phenotypic analysis of immune cells residing within the interstitium or epithelia of the oral mucosa. Methods such as whole animal perfusion using saline postmortem to flush blood from tissues have had mixed success (28, 30). We determined that intravenous perfusion to remove leukocytes within capillaries was both time consuming and gave inconsistent results in the relatively small tissues of the oral mucosa (data not show). For these two reasons, we chose to adapt a method described by Anderson and colleagues that used intravenous delivery of anti-mouse CD8 mAb conjugated to fluorochromes to stain and exclude CD8 T cells residing in the blood from CD8 T cells residing in the lung tissue (28, 32).

**Figure 5:**
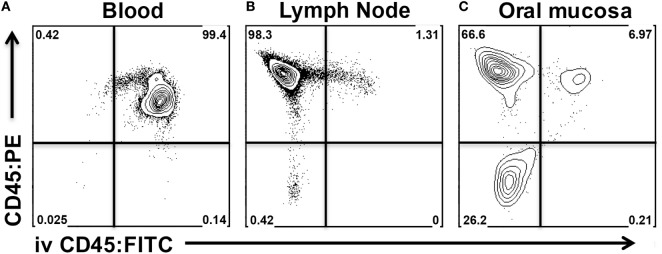
Intravenous anti-CD45 mAb distinguishes interstitial leukocytes from circulating leukocytes in oral mucosa. Anesthetized C57BL/6J mice were given 1.25 µg of FITC-conjugated anti-CD45 (CD45:FITC) mAb in 200 µl of PBS *via* retro-orbital intravenous injection. Mice were euthanized at 3 min. Single-cell suspensions obtained from blood, cervical lymph nodes (CLNs), and oral mucosa were stained with vitality dye Zombie NIR followed by rat anti-Mouse CD45 mAb conjugated to PE (CD45:PE). Cells were analyzed by flow cytometry. Dot plots from **(A)** blood, **(B)** CLN, and **(C)** oral mucosa samples gated on live cells obtained from a representative mouse are shown.

Our aim was to differentiate all leukocytes residing in the vasculature or in contaminating blood from those that have extravasated into the interstitium of the oral mucosa. For this purpose, anesthetized mice were injected intravenously with anti-mouse CD45 mAb conjugated to fluorescein isothiocyanate (CD45:FITC mAb) through the retro-orbital sinus. Mice were euthanized at 3 min post injection. Blood, CLNs, and oral mucosa were then harvested and prepared for analysis by flow cytometry following staining with anti-mouse CD45 conjugated to phycoerythrin (CD45:PE mAb). Due to intravenous delivery of non-saturating amounts of CD45:FITC mAb, we predicted two CD45^+^ population: CD45:PE^+^ CD45:FITC^+^ (circulating) and CD45:PE^+^ CD45:FITC^−^ (interstitial). We determined that a 3-min exposure to intravenous CD45:FITC mAb in a live mouse was sufficient to consistently stain greater than 99% of leukocytes in the blood (Figure 5A). Since intravascular free unbound CD45:FITC mAb could potentially stain cells during the harvesting and processing of the oral mucosa, we measured the extent of CD45:FITC staining on cells recovered from CLNs as a surrogate tissue sample that is vascularized and highly populated. Greater than 98% of the leukocytes from CLN samples were CD45:PE^+^ but CD45:FITC^−^ indicating that leukocytes were not stained by free CD45:FITC mAb during tissue processing (Figure 5B). The small number of double positive (CD45:PE^+^ CD45:FITC^+^) leukocytes is to be expected and can be attributed to either blood contamination of the CLNs during dissection or to cells residing in the vasculature of CLNs. Next, we examined oral mucosa samples recovered from the same mouse (Figure 5C). Here, we found that 66.6% of CD45^+^ cells that did not stain with CD45:FITC mAb (Figure 5C upper left quadrant). Those CD45^+^ cells had presumably extravasated out of the blood capillaries that supply the oral mucosa and migrated into the interstitium, shielding them from the intravascular CD45:FITC mAb. These cells are true interstitial leukocytes. The CD45:PE^+^ CD45:FITC^+^ leukocyte population recovered from the same sample (Figure 5C upper right quadrant) are leukocytes inside vessels of the oral mucosa or originating from blood released during dissection.

**Figure 6:**
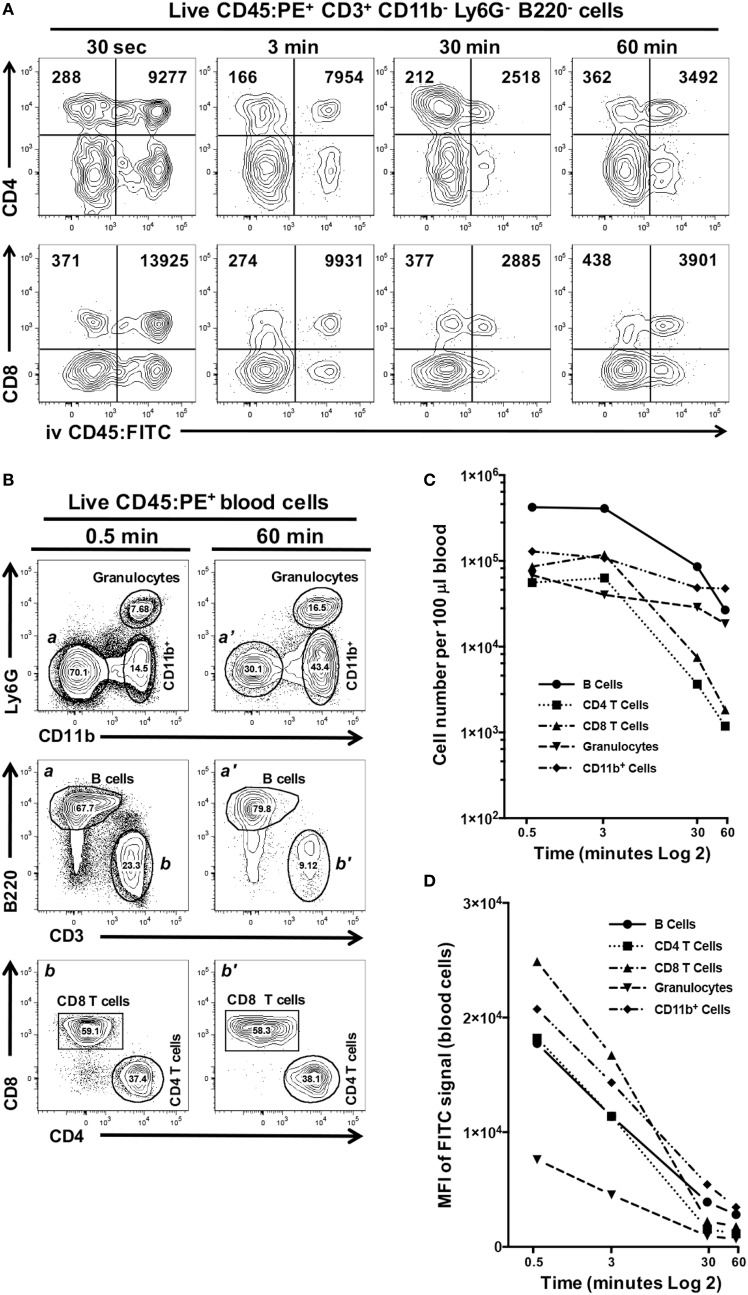
Increased exposure time to intravenous anti-CD45 mAb results in poor discrimination between interstitial and circulating leukocytes. Anesthetized C57BL/6J mice were given 1.25 µg of CD45:FITC mAb in 200 µl of PBS *via* retro-orbital intravenous injection and mice were euthanized at 0.5, 3, 30, or 60 min. Separate blood and oral mucosa samples were pooled from three mice. Processed single-cell suspensions were stained with vitality dye Zombie NIR followed by rat anti-mouse CD45:PE, B220c, CD3, CD4, CD8, CD11b, and Ly6G mAbs to identify live immune cell subsets in blood and oral mucosa samples. **(A)** Representative dot plots are shown of live CD4 or CD8 T cells identified from mucosal samples taken at 0.5, 3, 30, or 60 min. Numbers in the upper two quadrants indicate the mean fluorescence intensity (MFI) of the FITC signal found on cells within those quadrant populations. **(B)** Identification of CD11b^+^ cells and granulocyte, B cell [gates and panels *a* and *a’*], CD4 T cell, CD8 T cell populations [gates and panels *b* and *b’*] in pooled blood samples taken from mice at 0.5 or 60 min. Percentage of each population within a gate is given. **(C)** Number of live B cell, CD4 T cell, CD8 T cell, CD11b^+^, and granulocyte populations identified in 100 µl of blood samples taken at 0.5, 3, 30, or 60 min. **(D)** MFI of FITC signal found in the B cell, CD4 T cell, CD8 T cell, CD11b^+^, and granulocyte populations identified in 100 µl of blood samples taken at 0.5, 3, 30, or 60 min.

We next examined if increasing the exposure time to CD45:FITC mAb in a live mouse would result in better discrimination between interstitial and circulating leukocytes. In this experiment, we compared exposure times of 0.5, 3, 30, and 60 min and calculated the mean fluorescence intensity (MFI) of the CD45:FITC signal on CD4^+^ and CD8 T cells isolated from oral mucosa (Figure 6A). We observed that 3 min gave the clearest separation between CD45:PE^+^ CD45:FITC^−^ interstitial leukocytes and CD45:PE^+^ CD45:FITC^+^ circulating leukocytes, in both the CD4 and CD8 T cell populations. We found 3 min to also give the best discrimination between interstitial and circulating populations of B cells, granulocytes and CD11b^+^ monocytes/macrophages (data not shown). We observed only a slight increase in the background MFI of the FITC signal on CD45:PE^+^ CD45:FITC^−^ cells over time, indicating that leakage of CD45:FITC mAb into tissues from the vasculature is negligible. However, we did see a remarkable decrease in the MFI of the FITC signal on circulating CD4 and CD8 T cells due to longer *in vivo* exposure to CD45:FITC mAb. For example, the MFI of the FITC signal on circulating CD8 T cells went from 13,925 at 0.5 min to 3,901 at 60 min (Figure 6A). The net result of this MFI decrease is reduced distinction between interstitial and circulating immune cell populations.

We examined this phenomenon further by comparing the number of granulocytes, B cells, CD4 T cells, CD8 T cells, and CD11b^+^ cells found in blood samples from these mice obtained at the time of sacrifice (representative flow cytometry plots for 0.5 and 60 min time points are shown in Figure 6B). Increasing the exposure of mice to the CD45:FITC mAb resulted in a dramatic decrease in the number of live leukocytes (Figure 6C). CD4 and CD8 T cell populations showed the biggest decline. Moreover, all five leukocyte subsets examined also showed considerable decreases in the MFI of the FITC signal by 60 min (Figure 6D), consistent with what we observed in the mucosal tissue samples from these mice.

### Antigen-Experienced Interstitial CD4 T in the Oral Mucosa

**Figure 7:**
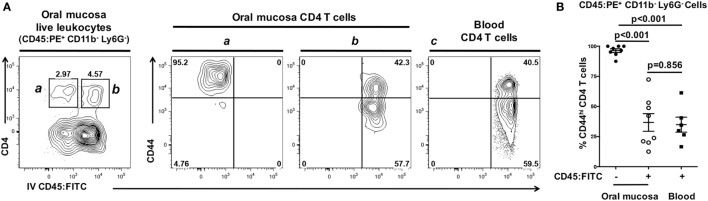
Interstitial CD4 T cells from the oral mucosa have a CD44^hi^ phenotype. Anesthetized C57BL/6J mice were given 1.25 µg of FITC-conjugated anti-CD45 (CD45:FITC) mAb in 200 µl of PBS *via* retro-orbital intravenous injection. Mice were euthanized at 3 min. Single-cell suspensions obtained from blood were stained with vitality dye Zombie NIR followed by rat anti-mouse CD45:PE, B220, CD3, CD4, CD8, CD11b, Ly6G, and CD44 mAbs to identify CD4 T cell populations. Cells were analyzed by flow cytometry. **(A)** Dot plots from blood and oral mucosa samples gated on live Ly6G^−^ CD11b^−^ leukocytes (CD45:PE^+^) from a representative mouse are shown. In oral mucosa samples, CD4 T cells were identified as interstitial (CD4^+^ CD45:FITC^−^) denoted by the *a* gate and contour plot; or circulating (CD4^+^ CD45:FITC^+^) denoted by the *b* gate and contour plot. Circulating (CD4^+^ CD45:FITC^+^) CD4 T cells in blood are bimodal with high and low CD44 expression (panel *c*). Percentage of cells within a drawn gate is given. **(B)** Summary data of the% of antigen-experienced (CD44^hi^) CD4 T cells found in blood, or in the interstitial (CD45:FITC^−^) or circulating (CD45:FITC^+^) compartments of the oral mucosa. Data are from two experiments of at least six mice and is displayed as mean ± SEM. Student’s two-tailed *t*-test was used to compare means.

CD45:FITC mAb delivered intravenously stained leukocytes within the bloodstream but not leukocytes within the interstitium or epithelium of the oral mucosa. To scrutinize this finding, we examined expression of CD44 as a marker of activation and antigen-experienced status in CD4 T cells. When examining CD44 expression on CD45:FITC^−^ (negative = interstitial) and CD45:FITC^+^ (positive = circulating) CD4 T cell fractions from oral mucosa, nearly all CD45:FITC^−^ CD4 T cells expressed high levels of CD44 (Figure 7A—gate and panel a). In contrast, CD45:FITC^+^ CD4 T cells showed bimodal expression of CD44 (Figure 7A—gate and panel b) matching the CD44 expression in a blood sample taken from the same animal (Figure 7A—gate and panel c). In the CD45:FITC^−^ fraction of the oral mucosa the frequency of CD44^hi^ CD4 T cells was significantly higher than in the CD45:FITC^+^ fraction of oral mucosa or blood cells (Figure 7B). Therefore, as predicted the extravasated interstitial CD4 T cells residing in the oral mucosa are almost all CD44^hi^, indicative of an antigen-experienced phenotype, whereas those identified as being from blood in oral mucosal samples are a mix of recirculating naïve CD44^lo^ and antigen-experienced CD44^hi^ CD4 T cells.

### The Relative Frequency of B Cells and Granulocytes in the Interstitium and Epithelium of the Oral Mucosa Can Be Skewed by Unaccounted Circulating Leukocytes

**Figure 8:**
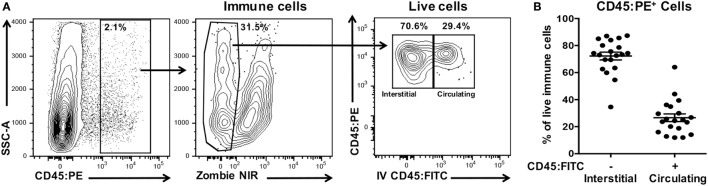
Processed oral mucosa samples are contaminated with on average 25% circulating leukocytes. Anesthetized C57BL/6J mice were given anti-CD45:FITC mAb as described in Figure 7 and euthanized at 3 min. Single-cell suspensions were processed from oral mucosa samples from single mice, stained with vitality dye Zombie NIR followed by rat anti-mouse CD45:PE to identify live immune cells by flow cytometry. **(A)** Gating strategy used to identify live interstitial (Zombie NIR^low^, CD45:PE^+^, CD45:FITC^−^) and circulating (Zombie NIR^low^, CD45:PE^−^, CD45:FITC^+^) immune cell populations. Percentage of cells within a drawn gate is given. **(B)** Distribution of immune cells from oral mucosa samples into interstitial or circulating leukocytes fractions. Data are from three independent experiments (*n* = 20).

We have established a simple and robust method to distinguish leukocytes infiltrating the interstitia and epithelia from those residing in extravascular blood or trapped within the vasculature of the excised oral mucosae at the time of harvest. Next, we determined the extent of potential contamination by circulating CD45^+^ leukocytes in oral mucosal samples (Figure 8A) and determined that these contaminants ranged from 10 to 65% of the total leukocyte population (Figure 8B). The result of not excluding these contaminating cells would be a significant overestimation of the number and phenotype of interstitial leukocytes in the oral mucosa.

**Figure 9:**
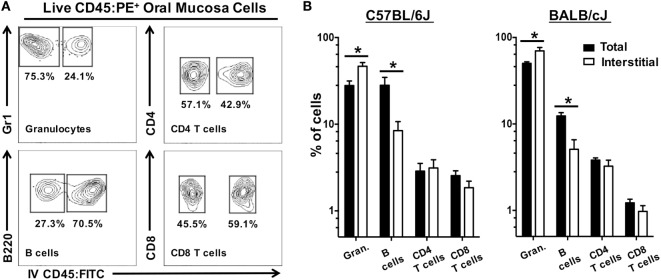
Unaccounted circulating leukocytes skew frequency of granulocytes and B cells in oral mucosa samples. Anesthetized C57BL/6J or BALB/cJ mice were given anti-CD45:FITC mAb as described in Figure 7 and euthanized at 3 min. Oral mucosa were pooled from two mice and processed as single-cell suspensions. Cells were stained with vitality dye Zombie NIR followed by rat anti-mouse CD45:PE, CD3, CD8, CD4, B220, and Gr1 mAbs to identify live leukocyte cell subsets by flow cytometry. **(A)** Gating strategy used to identify live interstitial (Zombie NIR^low^, CD45:PE^+^, CD45:FITC^−^) and circulating (Zombie NIR^low^, CD45:PE^−^, CD45:FITC^+^) granulocytes (Gr1^+^, B220^−^, CD3^−^), B cells (B220^+^, Gr1^−^, CD3^−^), CD4 T cells (CD3^+^, CD4^+^, B220^−^), and CD8 T cells (CD3^+^, CD8^+^, B220^−^). Percentage of cells within a drawn gate is given. **(B)** Frequency of granulocytes, B cells, CD4 T cells, and CD8 T cells found in oral mucosa samples as a percentage of total immune cell populations (both circulating and interstitial) or interstitial immune cell populations alone. Data are displayed as mean ± SEM. Student’s two-tailed *t*-test was used to compare means (*p < 0.05, *n* = 4).

We next examined if CD45:FITC^+^ circulating leukocytes would also affect the frequency of leukocytes subsets within the interstitia and epithelia of the oral mucosa. Frequency, rather than absolute number, is often used as a metric in analysis of tissue leukocytes. The frequency of granulocytes (CD45^+^, CD11b^+^, Gr1^+^, B220^−^, CD3^−^), B cells (CD45^+^, B220^+^, CD11b^−^, Gr1^−^, CD3^−^), CD4 T cells (CD45^+^, CD3^+^, CD4^+^, CD8^−^, CD11b^−^, Gr1^−^, B220^−^), and CD8 T cells (CD45^+^, CD3^+^, CD8^+^, CD4^−^, CD11b^−^, Gr1^−^, B220^−^) was determined in total CD45:PE^+^ leukocytes (CD45:FITC^+^ and CD45:FITC^−^) and interstitial CD45:PE^+^ leukocytes (CD45:FITC^−^) (Figure 9A). In both BALB/cJ and C57BL/6J mice, we determined that the frequency of interstitial granulocytes (CD45:FITC^−^) would be significantly under estimated, while the frequency of interstitial B cells (CD45:FITC^−^) would be significantly over estimated if CD45:FITC^+^ (circulating) cells were not excluded from the analysis (Figure 9B). Taken together, unaccounted and non-excluded circulating leukocytes will result in an overestimation of extravasated interstitial leukocytes and a biasing of the relative frequencies of a number of immune cell types present in the extravascular tissue compartment of the oral mucosa.

## Discussion

Here, we describe a simple robust method that combines intravenous delivery of a pan-leukocyte (CD45) monoclonal antibody with a flow cytometry approach to discriminate interstitial leukocytes in the oral mucosa from those found within blood; either residing within the tissue vasculature itself or in blood dispersed during dissection that could contaminate the surface of the mucosal tissue. Although histological techniques have been useful in identifying a number of cell types within the oral mucosa of mice and its vasculature system (45, 46), histological techniques do not have the same quantitative and qualitative accuracy offered by flow cytometry-based methods (10, 47). For example, we have used the method described here to phenotype, based on cytokine production, CD4 T cells that have extravasated into the interstitium of the oral mucosa of mice (Bittner-Eddy et al., unpublished data). Moreover, the method described here offers the advantage of simultaneously discriminating the interstitial cells from the leukocytes residing within the vasculature either as part of the recirculating component or as cells that have adhered to activated luminal endothelial cells. Notably, these adherent cells are not removed in procedures that employ vascular perfusion postmortem (31).

We show that circulating leukocytes can represent a significant source of contaminating leukocytes in oral mucosa samples processed for flow cytometry. If unaccounted for, the circulating leukocytes would not only inflate the numbers of “tissue”-infiltrated leukocytes but would also potentially bias the relative frequencies of a number of key leukocyte subsets. A significant number of publications examining immune cell content of the gingiva of mice under inflammatory conditions have not taken account of the potential for blood contamination (10, 21, 22, 47–49). It is well established that key immune cells involved in inflammatory responses in tissues have very different phenotypes and activation states compared to those found in the bloodstream (24–27). As further evidence of this fact, we show here that CD45:FITC^−^ CD45:PE^+^ CD4 T cells identified in the interstitium of the oral mucosa almost universally have a CD44^hi^ phenotype, which is a classic marker of antigen-experienced CD4 T cells (50). These CD4 T cells have very specific activation states including expression of specific cytokines that differ from naïve circulating CD4 T cells. When tissue-infiltrating antigen-specific memory CD4 T cells are rare, it is difficult to distinguish them from the “noise” of contaminating cells of a similar lineage. The consequence of not excluding these contaminating cells is even more significant when analyzing extremely small tissue specimens like the murine gingiva.

While optimizing the method of anti-mouse CD45 mAb we also found that extending the intravenous exposure time beyond 3 min resulted in fewer live cells being recovered from the oral mucosa samples. Anti-CD45 mAbs have been used on human leukocyte cell lines and intravenously in rats as a means to deplete hematopoietic cells (51–54). The depletion of CD45^+^ cells when the anti-CD45 mAb persists more than 3 min can be attributed to complement dependent cytotoxicity and/or antibody-dependent cell-mediated cytotoxicity. Interestingly, the only other reported use of anti-mouse CD45 mAb to stain mouse leukocytes *in vivo* was with 10 µg of mAb and an exposure time of 5 min (31). These authors did not specifically look at cell death, but did not report any issues in their analysis of leukocytes in lung tissues. We also observed a noticeable decrease over time in the MFI of the FITC signal on all live leukocyte subsets examined. This affect may in part be due to the selective killing of cells with more CD45 bound by mAb or receptor internalization and degradation. It is interesting to note that CD8 T cells had the highest FITC MFI presumably through higher cell surface expression of this protein and it is CD8 T cells that showed the most rapid decrease in MFI of the FITC signal over the 60 min interval. Wulf et al. (53) reported differential cytolytic activity of anti-CD45 mAb on different human cell lines, although they did not correlate this effect with CD45 expression levels (53). Nonetheless, time of exposure and dose of mAb are important parameters to consider so as to maximize discriminatory power and minimize selective loss of cell vitality.

We also describe a very detailed dissection protocol to avoid contamination with NALT, a secondary lymphoid tissue located immediately cranial to the anterior hard palate. NALT contamination of dissected oral mucosa resulted in inflated lymphocyte numbers, particularly B cells and can give misleading results if not avoided. Several investigators have reported splitting of the maxilla in order to isolate oral mucosal tissues such as the marginal gingiva and these samples may have been unintentionally contaminated with lymphocytes from the NALT (10, 21–23). Avoiding the NALT is critical to any assessment of oral mucosal tissues that contain low numbers of extravasated lymphocytes. This statement is underlined by our observation that *Pg*-specific antigen-experience CD4 T cells could be recovered from the NALTs of mice that had been orally fed *Pg*. It is known that NALT can act as a major mucosal inductive site that provides both B and T memory cells for local dissemination (42) and the intranasal delivery of *Streptococcus pyogenes* results in a robust Th1 (36) and Th17 memory response in mice (43, 44). Our data do not indicate whether or not the NALT is acting as an inductive site or whether we are detecting *Pg*-specific CD4 T cells that are circulating between the oral mucosa and the NALT. We consider the latter unlikely because we observed higher frequencies of *Pg*-specific antigen-experience CD4 T cells in the NALT compared to the CLNs that drain the oral mucosa (17). Certainly it is possible for mice to acquire *Pg* directly into their nasal passages *via* the patent nasopalatine canal during nuzzling activity (39–41). This activity ultimately provides the opportunity for *Pg* antigens to be directly presented to naïve CD4 T cells in the NALT.

Notwithstanding improvements in techniques for the recovery of live cells from oral mucosa (20) or novel methods of recovering lymphocytes from gingival tissue that avoid enzymatic digestion (55), researchers still need to account for blood-borne cells, and most importantly, to avoid NALT contamination of oral mucosal tissue when working with mouse models of periodontitis. The novel method described here allows the quantification and analysis of true interstitial leukocytes in the oral mucosa at homeostasis or following inflammation induced by mucosal colonization with a pathogen like *Pg*.

## Ethics Statement

This study was carried out in accordance with the recommendations of the Association for Assessment and Accreditation of Laboratory Animal Care. The protocol was reviewed and approved by the Institutional Animal Care and Use Committee of the University of Minnesota (Protocol ID # 1511-33178A).

## Author Contributions

PB-E: intellectual contribution of the design of most experiments and performed all flow cytometry investigations and drafted the first version of the manuscript. LF: maintained the murine colony, cultured *Pg*, and conducted all murine oral inoculations as well as intellectual contribution. AT: in cooperation with PB, isolated and purified the R/Kgp tetramer with intellectual contribution. DA: performed initial perfusion experiments and initial isolations of cells from oral mucosa with intellectual contribution. MC: intellectual contribution to the design and troubleshooting of all experiments, edited the final version of the manuscript and performed the dissection of all murine oral mucosa specimens and NALT.

## Conflict of Interest Statement

The authors declare that the research was conducted in the absence of any commercial or financial relationships that could be construed as a potential conflict of interest.
