# Supplementary figures and images for: Discriminating between Interstitial and Circulating Leukocytes in Tissues of the Murine Oral Mucosa Avoiding Nasal-Associated Lymphoid Tissue Contamination
Source: Front Immunol. 2017 Oct 30;8:1398. doi: 10.3389/fimmu.2017.01398 (PMC5666297; doi:10.3389/fimmu.2017.01398)

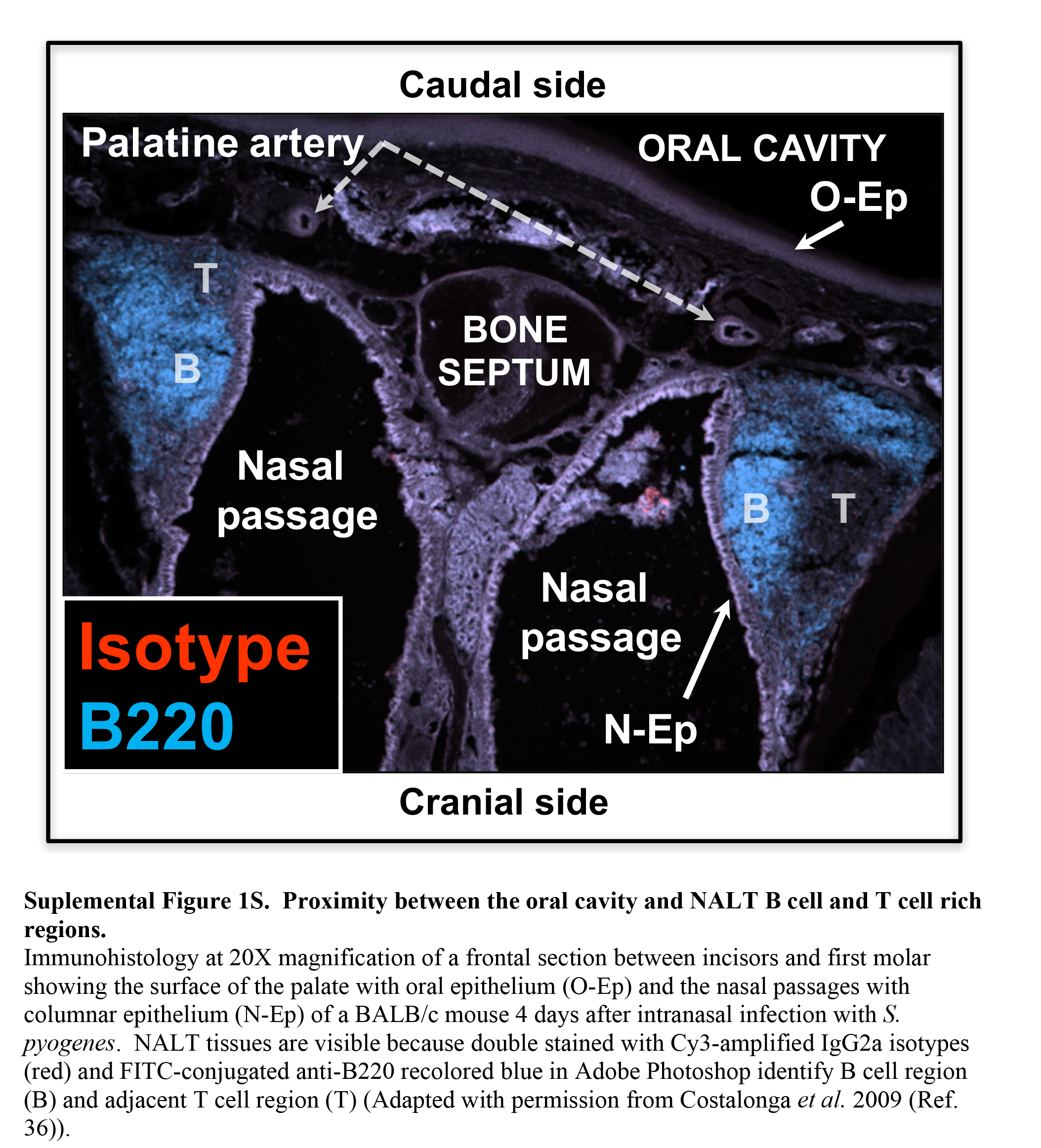

Supplement: Supplementary file 1 [file Image_1.tif]
